# Green space for public mental health: an ethnographic study of ecotherapy in Wales

**DOI:** 10.1177/17579139231170777

**Published:** 2023-05-02

**Authors:** E Lord

**Affiliations:** Swansea University, Glyndwr 214, Swansea SA2 8PP, UK

**Keywords:** mental health, green space, ecotherapy, ethnography, health inequalities, social prescribing

## Abstract

**Aims:**

In recent years, there has been a growing interest in the ways that human health intersects with exposure to nature. This article reports the findings of a research study investigating the experiences of people in South and West Wales who were engaged in a specific type of nature and health intervention: ecotherapy.

**Methods:**

Ethnographic methods were used to develop a qualitative account of the experiences of participants in four specific ecotherapy projects. Data collected during fieldwork included notes from participant observations, interviews with both individuals and small groups, and documents produced by the projects.

**Results:**

Findings were reported using two themes: ‘smooth and striated bureaucracy’ and ‘escape and getting away’. The first theme focused on how participants negotiated tasks and systems related to gatekeeping, registration, record keeping, rule compliance, and evaluation. It was argued that this was experienced differently along a spectrum between striated, in which it was disruptive to time and space, and smooth, in which it was much more discrete. The second theme reported on an axiomatic perception that natural spaces represented an escape or refuge; in terms of both reconnecting with something beneficial in nature, and also disconnecting from pathological aspects of everyday life. In bringing the two themes into dialogue, it could be seen that bureaucratic practices often undermined the therapeutic sense of escape; and that this was more acutely experienced by participants from marginalised social groups.

**Conclusions:**

This article concludes by reasserting that the role of nature in human health is contested and arguing for a greater emphasis on inequities in access to good quality green and blue space. Specific interventions like ecotherapy need funding models that avoid striated bureaucratic processes, and the stress associated with these. Inclusive models of ecotherapy practice could contribute to public health goals related to population engagement with healthy environments.

## Introduction

This article reports the findings of a research study investigating the experiences of people in South and West Wales who were engaged in a health and wellbeing intervention called ‘ecotherapy’. Using ethnographic methods, including participant observation, interviews, and analysis of documents, between 2017 and 2020, this study focused on four different projects that met a definition of ecotherapy used by the UK mental health charity Mind:

*‘Ecotherapy (sometimes called green care) comprises nature-based interventions in a variety of natural settings. Ecotherapy initiatives usually consist of a facilitated, specific intervention’.* ^1^

It was surmised early in the study planning stage that these four projects were local manifestations of a much wider trend, seen in multiple places globally. This wider trend can be summarised as a growing interest in the ways that human health intersects with exposure to nature, an interest that is observable in practical applications,^2,3^ research activity,^4^ institutional reports,^5^ and references in popular culture.^6,7^ It has been argued elsewhere^8^ that this nature and human health theme can be seen as a cultural zeitgeist in numerous global contexts.

While the nature and health trend has many manifestations globally, it arguably reached a greater level of public and professional visibility in the UK when the prominent mental health charity ‘Mind’ launched their ‘Ecominds’ project in 2007. In this initiative, 130 projects in England collectively labelled as ‘ecotherapy’ were funded (with a National Lottery grant of £7.5 million) for a period of 5 years from 2008 to 2013. The rationale for Mind to launch the Ecominds project was described in Bragg et al.’s evaluation report as a response to the need to find a solution which could simultaneously address both the cost challenges of mental healthcare, and the need for increased service accessibility for a diversity of people, this is summarised: ‘There is now more need than ever to explore different preventative and curative therapies to add to the “toolbox” of treatment options’.^1^

This ecotherapy intervention strand of the wider nature and health domain can thus be seen as strongly intertwined in the politics, policy imperatives,^9^ and contestations of mental health service provision, including the so-called polyvalence of the recovery concept.^10^ It is also closely allied to government attempts in some nations to embed, in multiple sectors, wellbeing outcomes intended to improve population health across the lifespan^11^; a policy orientation exemplified in Wales by the Wellbeing of Future Generations (Wales) Act (2015) devised by the devolved government. Bragg et al.^1^ pointed to the increasing research evidence in the nature and health domain, and also, the increase in programmes from government and third sector bodies to increase engagement with nature, but suggested that ecotherapy interventions lack a broad credibility among key stakeholders:

*‘It is apparent that there is an emerging body of evidence supporting green exercise and ecotherapy and it is becoming increasingly recognised as an idea which can be linked to current government health and social care policies. However there is still a way to go before ecotherapy is considered “mainstream” as a way to increase wellbeing or as a treatment option in mental healthcare’.* ^1^

The impetus for this research study came from reflecting on this notable increase in the prevalence of ecotherapy initiatives and interventions, alongside the complex ways they were negotiating both the contested field of mental health service provision and wider wellbeing policy and practice.

Research into the connections between nature and human health has been greatly expanding over the past two decades, as noted in a number of reviews.^4,12,13^ However, it is reasonable to say that much of this research effort is focused on identifying pathways and mechanisms at both individual and population levels, typically with positivist assumptions and relying on biomarkers and other reified measurable factors (these methods and some of the potential instrumental effects of this focus have been critiqued in greater detail elsewhere).^14^ In contrast, this study was focused on gaining some understanding of what ecotherapy meant to participants, and those delivering the interventions, and specifically, how they saw its interface with what are commonly seen as more mainstream mental health services and interventions. Specifically, one of the objectives was to explore whether it was seen by those involved as either oppositional or adjunctive to mainstream services; and why it often remains implicit and unarticulated whether ecotherapy is intended as an intervention for a clinical population or a more general preventive public health opportunity.

## Methods

Early in the study planning process ethnography was identified as congruent with the study’s concerns around constructing situated^15^ and non-reductive data about the ecotherapy field.^16^ While much of the research in the nature and human health field is founded on (usually implicit) positivist assumptions, the use of ethnographic methods in this instance is based on an explicit constructionist assertion^17^ that research data are always already imbricated within complex social fields.^18^ This methodological approach builds on assertions made by O’Brien and Varley^19^ about the valuable applications of ethnography to the empirical understanding of human engagement with nature.

In this research, hard definitions were not applied to either what a natural space or place is or to terms like ‘mental health’ and ‘wellbeing’. Instead, the definitions of nature and health/wellbeing that were being explicitly articulated or used tacitly by those in the field were sought. This is coherent with an ethnographic approach to research^20,21^ and avoids the pitfalls of trying to measure or reify either of these contested domains.

Three specific types of data were collected during fieldwork: notes from participant observations, recorded and transcribed interviews with both individuals and small groups, and documents produced by the projects; although these three should not be seen as fixed, and distinct categories as fundamentally ethnography is concerned with the integration of – and dialogue between – multiple data types. To give an indication of the amount of data produced; 450 hours were spent as a participant observer directly engaged in fieldwork within the selected projects.

A purposive approach to sampling^22^ was employed, in terms of identifying projects that met the definition of ecotherapy within the geographical area under review. The final four projects in which fieldwork was conducted were an off-road running group: *Trail Runners*, a sustainability skills organisation: *Planet4People*, and two woodland-based interventions: *WellWoods* and *EcoConnect* (all project and individual names are pseudonyms to protect participant anonymity). It was interesting to note that the interventions offered by these projects were largely aimed at non-clinical populations, and, other than for pragmatic recruitment issues when they were marketed to specific groups, it was mostly left to participants to decide on their need, attendance, and anticipated outcomes. A further pertinent observation was that these projects were all staffed by non-healthcare professionals, and, in most instances, they could be considered as peer-to-peer; in terms of being managed and run by people with a personal experience of, and passion for, wellbeing in nature practices. The main interface with statutory health services was via a variety of makeshift, informal, and piecemeal referral practices, some of which were under review by the projects with the aim of making standardised social prescribing packages in the future. This again points to the emergence of new ways of working, like social prescribing, that are associated with broader population wellbeing discourses.

Ethical approval was sought from the Research Ethics Committee embedded within the Swansea University College of Human and Health Sciences; permission to proceed was granted by this committee in May 2017. This included ensuring all participants had clear information about the study, what data were being collected, how it was managed, and were given sufficient notice and opportunity to opt out of participating in the study.

Data analysis was accomplished by multiple stages of qualitative coding, using what Lune and Berg^23^ liken to a funnel shape. This started by making analytic notes during fieldwork and assigning open codes to fieldnotes, interview transcripts, and project documentation. In most forms of ethnography, there is an iterative process of learning and modification going on throughout and data analysis is not a separate and discrete stage in a linear process but is inter-leafed with ongoing decisions about data collection.^21,24^ From this open coding phase a total of 80 codes were devised, comprised of single words or short phrases. In the next phase of analysis, bearing in mind the funnel analogy,^23^ this lengthy list of 80 codes was reduced (funnelled) into fewer categories by reflecting on linkages, connections, and patterns, within and between the codes. This analytic process was informed by the ethnographic orientation of the study; in the broadest sense, this was about having an interest in interactions and negotiations between people, spaces, places, and cultural and institutional arrangements.^17,20,21^ More specifically, the coding, funnelling, and theme construction involved identifying and interrogating the situated experience of ecotherapy as it was occurring in actual places, the meanings that were being attributed to it by people in these settings, and how these meanings informed its relations with other mental health technologies, services, and interventions. An example of this type of analysis was the identification of whether, or not, individual participants articulated an outcome they expected from the activity, and what the nature of this outcome was; the coding process helped to link these ideas of outcome to other factors, like what kind of activity was taking place, how the participant had ended up attending the project, and what other experiences and expectations they had of mental health and wellbeing interventions/services. By the end of the analysis process, two themes had been constructed: ‘*smooth and striated bureaucracy*’ and ‘*escape and getting away’*. By making the connections and links between codes, these themes then informed a rich, detailed, and credible ethnographic account of the experiences and construction of the ecotherapy ‘field’ in these four projects at this time.

## Results

The first theme was called ‘smooth and striated bureaucracy’, and this focused on the organisational systems deployed within the four ecotherapy projects and, specifically, how these were negotiated by participants. It is argued that what was of particular note within this theme was the ‘point of suture’ between abstract ‘external’ bureaucracy, and immanent activity ‘internal’ to the field. Activity related to organisational systems is common in contemporary life, a point evocatively summed up by Graeber’s^25^ suggestion that ‘bureaucracy has become the water in which we swim’. The ecotherapy field is no exception to this bureaucratic trend ‘that is such a pervasive feature of modern social institutions’,^20^ and the construction, accumulation, and sharing of standardised data was a distinct set of tasks achieved in some fashion by all of the projects in this study.

The deployment of bureaucratic tasks and the different strategies of engagement with, avoidance of, and resistance to these tasks was a notable part of many of the observational periods in the field. This is a facet of ecotherapy that is largely lacking analysis in the existing research literature, but its prominence in this research fieldwork was striking. Specifically, it is argued that these tasks could be seen on a spectrum between ‘smooth’ and ‘striated’ – the smooth being discrete and hard to even notice, while the striated required disruptive use of time, space, and attention. A strength of ethnography is that it brings together multiple types of research data to indicate the negotiations that go behind the polished ‘finished product’ of bureaucracy that may be publicly available – the kind of presentation that an organisation would publish on its website or in a report to funders.^26,27^ As Atkinson^20^ reminds us: ‘Organisational records do not necessarily provide transparent representations of ‘what happened’’, while documents may be ‘invoked to justify and legitimise courses of action’, it can be widely observed in a plethora of organisational fields that the actors creating these documents rarely ‘follow bureaucratic rules to the letter’. Thus, in analysing the research data from the ecotherapy field, critical questions were posed of how the production of gatekeeping, registration, record keeping, rule compliance, and evaluation data were being negotiated. The analysis process included identifying what the stated purposes of the information gathered by the projects was, what strategies were put in place to facilitate the collection of these different types of data, and how actors in the field were complying with or resisting this process.

This first theme establishes some of the key social processes, including the power relations embedded in these processes, that contribute to producing the ecotherapy space. Bureaucratic systems rely on assumptions of universalism, rationalism, and objectivity, alongside practices of abstraction, standardisation, commensuration, and reduction.^28,29^ Through these assumptions and practices, the ecotherapy field was anticipated by ‘external’ parties (including funders and referrers) to be equipped to deliver a set of measurable outcomes. This process, however, struggled to account for the nuanced and complex ways that wellbeing was experienced from an ‘internal’ (to the field, as well as the individual) perspective. Furthermore, there were actively negative consequences of the tasks that were initiated at the ‘point of suture’ between the ‘external’ and the ‘internal’. Specifically, it was found that already marginalised populations, such as a group of asylum seekers and a group of individuals with what could be considered serious psychiatric diagnoses, experienced the more striated gatekeeping, and evaluation tasks, compared to groups composed mostly of individuals from more privileged socio-economic groups, who’s experience was much more smooth. These more privileged groups included professionals who participated in the trail running groups; and the smooth bureaucracy – such as demonstrating outcomes by sharing attractive images of the activity on social media – was partly facilitated by the use of subscription funding models rather than the block grants other projects relied upon.

These findings indicate that these were important factors in how participants accounted for the wellbeing effects of ecotherapy, what the embodied and sensory experiences of these interventions were, and how likely it would be for natural spaces to be accessed for lifespan population wellbeing by groups who were not already regularly using these spaces. As an illustration of this; it was found that many of the participants benefitting from the smooth bureaucracy would reminisce about childhood experiences of nature, and lived in neighbourhoods closer to safe and attractive green space, this led to a familiarity and confidence with using green and blue spaces. One participant, *Archie*, was a healthcare professional participating in *Trail Runners* groups; he described nature as ‘like PRN’ which is the abbreviation of the Latin term for ‘as needed’ medication used in healthcare records. This indicated a feeling of agency in knowing what nature could offer, knowing when this was needed, and being able to access the requisite spaces and places in a timely fashion. Contrast this with another participant, *Grace*, who was referred by a local refugee and asylum seeker support organisation. *Grace* was from a minority ethnic background, had only recently moved to this part of Wales, spoke English as a second language, had sole caring responsibility for children, and was housed in a more deprived part of the city with limited access to safe green space. The group that *Grace* attended *Planet4People* with relied on pre-arranged mini-bus transport to attend the woodland site. During a fieldwork observation period, she discussed how much she loved these times in the woods and felt distracted from her everyday stress and anxiety during and after the ecotherapy sessions; however, she needed to miss some sessions because of childcare difficulties and was sad that she could not visit the woods or do the activities at another time. *Archie* and *Grace’s* experiences can be seen to represent the varied barriers and opportunities certain groups face at a population level, and the inequities around access to healthy spaces that are well documented in the public health literature.^30^

The second theme ‘escape and getting away’ relates to a widely held axiom that there is, or at least should be, something ‘different’ about so-called natural spaces such as woodland – an axiom that is commonly expressed linguistically in terms like ‘escape’, ‘refuge’, ‘freedom’, and ‘getting away’, and behaviourally in practices of exploration, expression, and playfulness. This notion is well summed up in this interview extract by *Pete*, a leader and participant in the *EcoConnect* project:

*‘a very positive, a very restorative, er . . . a very healing effect . . . particularly in the early years [of his mental health problems] as a place of sanctuary as well . . . a retreat . . . a retreat from the busyness of the world a retreat from things which were . . . which were causing my mental wellbeing to dip . . . so I . . . I always knew that I could retreat into nature and it was a comfort . . . it was a comfortable healing place to go . . .*’

In this extract, *Pete* deploys multiple terms in a short time to express the character he perceived in natural spaces at a period of crisis in his mental health. In his experience, being in nature was about *getting away* from the *causes* of his distress, to remain in that ‘restorative’ and ‘healing’ space as a ‘retreat’ and a ‘sanctuary’ for as long as he needed to, and, finally, to know that he could return as and when it was required. The process of ‘getting away’ was expressed, by many participants across all the projects, in terms of both ‘reconnecting’ with something within nature that had been lost or obscured, and also ‘disconnecting’ from something pathological/unhealthy within the more typical spaces of everyday life.

This expectation of what could be gained from natural spaces – in terms of both ‘reconnecting’ and ‘disconnecting’ – in the ecotherapy context is a reason why the first theme was of such significance. The striated bureaucratic processes seemed like an imposition: this was exactly the kind of thing participants needed to disconnect from. The striated ways that the bureaucratic processes were experienced by marginalised groups replicated their, often traumatic, experiences of negotiating complex systems like health, welfare, and immigration institutions. Therefore, the refreshing and replenishing richness of the escape experiences provided by time in nature was partially undermined by meeting these ‘external’ requirements.

## Discussion and Conclusion

A focus of interest in this research study in the field of nature and health was how it was being formed in relation to differing research practices, academic disciplines, and the institutional arrangements of healthcare and public health that made up the local conditions of its operationalisation. The expected aims, objectives, and outcomes of interventions at the nature and health intersection, and, indeed, its practical format, are multiple and remain unsettled and contested. An example of this contestation is the open question of whether nature is a repository of health for clinical populations or for lifespan preventive public health (even though Mind’s ecotherapy definition appears to lean to the former, this research indicates the activity on the ground is not so clear cut).

Despite this contestation, in the research literature there is a widespread emphasis on how to measure nature exposure or test particular psychological or biological pathways and mechanisms.^14^ In designing this research study in an ethnographic fashion, it was acknowledged that this emphasis in much of the research effort has a reductionist and reifying effect; this is because it focuses attention on the individual human as organism and seeks to find testable and repeatable chains of causation for the salutogenesis available from nature. This effect leads to a lack of critical attention to the myriad irreducible experiences and complex negotiations of people currently taking part in interventions and activities that make up the *form* of the nature and health intersection in particular places.

Ecotherapy is a term that evades capture, it is a concept that ‘floats’ between practices, ideas, theories, empirical studies, and the institutions that give concrete form to these things. It is what has been called, in a sociological sense, a ‘polyvalent concept’^31^ – it is deployed by multiple different interest groups using varied implicit meanings, often as a ‘working misunderstanding’ in which differences are ‘collusively ignored or bracketed’.^10^ To study many interventions in the health field, a succinct definition, however, contested or problematic, would be available as a starting point. Succinct definitions of this type usually originate from institutional actors with a responsibility for matters related to funding, accountability, and quality assurance. Such actors include the National Institute for Health and Care Excellence (NICE) in the UK. A definition from a source like this would typically include indications for what groups or diagnosis the intervention was expected to have efficacy for, who would be qualified to deliver such a thing, and what kind of effects would be expected as an outcome, as well as economic appraisals aimed at those commissioning health services.^32^

Ecotherapy does not currently enjoy this status of endorsement from such bodies in the UK, this is something which makes defining and subsequent setting of parameters for empirical research difficult, but it can also be argued there is a rich and exciting sense of possibility in this messy and open field on the margins of the mainstream. This makes it available for those who would rather reside and find their healing outside of mainstream services – a possibility that may be foreclosed if it is taken into the core of the mainstream and guarded by a range of striated policies, practices and procedures. The reliance of many ecotherapy projects on enthusiastic individuals with lived experience rather than professional groups is an interesting point to note in this regard.

As indicated by the above references to the Mind Ecominds initiative there can frequently be found within this field an urgency to connect with the ‘mainstream’: to somehow legitimise nature as a tool for either population preventive health or more individual treatment interventions (or both). This objective, or its assumed urgency, is rarely questioned and is lacking the sustained critical attention that such an assertion requires. This research has provided some insights into the complexities of how this is being operationalised on the ground, and it points to some specific areas of enquiry that would benefit from further research.

If critical distance can be found from the assumption that ecotherapy needs to be made mainstream as fast as possible, then numerous questions can be posed. For example, how are mainstream mental health services defined and delineated, and by whom? What kind of inclusions and exclusions permeate this domain? In defining ‘mainstream’ it also becomes imperative to enquire what it means to be oppositional and on the edge of mainstream services. It is arguably not the case that a binary in-or-out status for ecotherapy is needed, or is even possible, but it is interesting to reflect on what the instrumental effects of either position are, or could be.
